# A New Method for In-Situ Characterization of Solid-State Batteries Based on Optical Coherence Tomography

**DOI:** 10.3390/s24082392

**Published:** 2024-04-09

**Authors:** Jinze Li, Tianhong Ma, Xin Liu, Jiawei Xi, Li Deng, Hao Sun, Yanxin Yang, Xiang Li

**Affiliations:** 1 School of Optoelectronic Engineering, Xidian University, Xi’an 710126, China; lijinze@xidian.edu.cn (J.L.); jiaweixi@stu.xidian.edu.cn (J.X.); ldeng@stu.xidian.edu.cn (L.D.); yangyanxin@stu.xidian.edu.cn (Y.Y.); xiangli@xidian.edu.cn (X.L.); 2 Haishan Industrial Development Corporation, Shijiazhuang 050200, China; 1405110255@stu.xidian.edu.cn; 3 School of Physics, Xidian University, Xi’an 710126, China; liuxin0327@stu.xidian.edu.cn

**Keywords:** solid-state battery, optical coherence tomography, in situ characterization, lithium dendrite growth

## Abstract

With the in-depth study of solid-state batteries (SSBs), various in situ and ex situ characterization technologies have been widely used to study them. The performance and reliability of SSBs are limited by the formation and evolution of lithium dendrites at the interfaces between solid electrodes and solid electrolytes. We propose a new method based on optical coherence tomography (OCT) for in situ characterization of the internal state of solid-state batteries. OCT is a low-loss, high-resolution, non-invasive imaging technique that can provide real-time monitoring of cross-sectional images of internal structures of SSBs. The morphology, growth, and evolution of lithium dendrites at different stages of cycling under various conditions can be visualized and quantified by OCT. Furthermore, we validate and correlate the OCT results with scanning electron microscopy (SEM) and XPS, proving the accuracy and effectiveness of the OCT characterization method. We reveal the interfacial phenomena and challenges in SSBs and demonstrate the feasibility and advantages of OCT as a powerful tool for in situ and operando imaging of battery interfaces. This study provides new insights into the mechanisms and factors that affect SSB performance, safety, and lifetime, and suggests possible solutions for improvement and application in the field of applied energy.

## 1. Introduction

Solid-state batteries (SSB) are promising candidates for next-generation energy storage devices due to their advantages of high energy density, long cycle life, and safety. However, one of the major challenges facing solid-state batteries is the formation and growth of lithium dendrites at the interface between the anode and the electrolyte. Lithium dendrites are needle-like structures that grow from the lithium metal anode during the charging and discharging processes. These dendrites can penetrate the solid electrolyte and cause short circuit or even fires. Therefore, for improving the performance and safety of SSBs, it is crucial to understand the mechanism and dynamics of lithium dendrite growth in SSBs, as well as find effective ways to suppress or eliminate them. SSBs use solid electrolytes instead of liquid or gel electrolytes, effectively preventing the leakage and combustion of electrolytes. Additionally, they enable the use of lithium metal as an anode material, significantly increasing the specific capacity and reducing battery weight.

To investigate the interfacial behavior of lithium metal in solid-state batteries, various characterization methods have been developed, including X-ray diffraction (XRD) [1], X-ray computed tomography (XCT) [2], scanning electron microscopy (SEM) [3], transmission electron microscopy (TEM) [4], etc. However, these methods have some limitations, such as requiring special sample preparation, causing damage to the samples, having low spatial resolution, or being unable to perform in situ measurements. Therefore, there is a need for a new method that can overcome these drawbacks and provide real-time, non-invasive, and high-resolution imaging of SSBs during normal operation.

**Figure 1:**
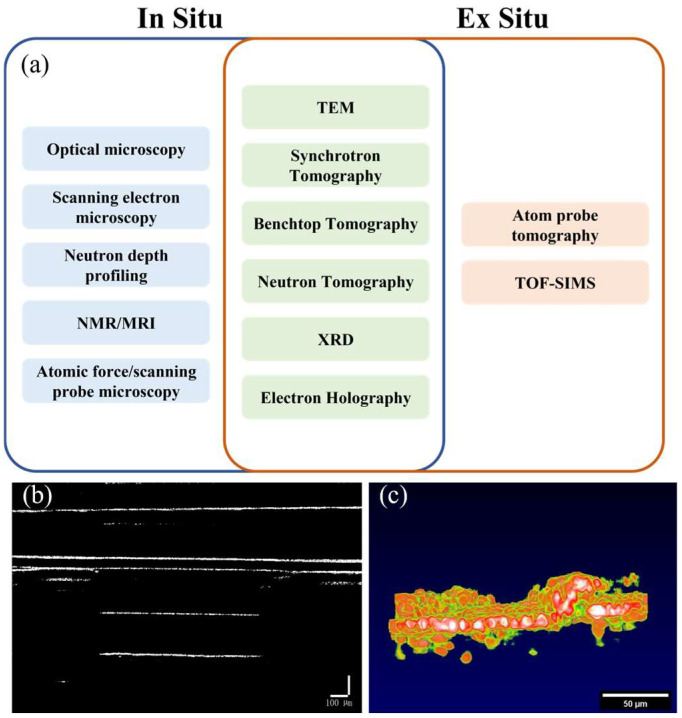
(**a**) In situ and ex situ monitoring methods for solid-state batteries [5,6,7,8,9,10,11,12,13,14,15,16,17,18,19,20], (**b**) two-dimensional image of battery interface, (**c**) two-dimensional side view image of battery interface.

SSBs exhibit multiple chemical, mechanical, and electrochemical transitions under both static and operating conditions. On the other hand, ex situ characterization can provide global information about system states and material transformations within SSBs, including a global understanding of degradation and failure mechanisms. However, in situ characterization is still required to understand the initiation and development of these mechanisms. Furthermore, the dynamic processes that occur in SSBs are often on extreme timescales. Under certain electrochemical boundary conditions, these processes are often difficult to capture under ex situ conditions. Therefore, in situ characterization is crucial for overcoming these limitations and enabling monitoring of the mechanisms under many specific conditions for solid-state batteries. Figure 1a shows most of the techniques currently used to monitor the mechanisms of solid-state battery static and operational processes.

Optical microscopy/SEM techniques have been used for planar/sectional imaging of SSB. TEM can obtain information related to material morphology and local atomic configuration through the generation of transmitted electrons. XRD is a scattering technique that detects the Bragg reflection peaks of ordered crystalline materials in a material system and provides information about the material structure. These monitoring methods currently in common use can either only perform two-dimensional monitoring (such as optical microscopy, SEM) or require complex and expensive equipment to achieve three-dimensional monitoring (such as TEM, XRD), as shown in Table 1. Although the current detection technology can realize the internal characterization of the battery, to detect the internal status of the battery while the battery is working, we propose a method of using OCT for in situ battery characterization. This method is expected to be used in the detection of solid-state batteries at lower costs and with higher efficiency in the future.

Optical coherence tomography [21] (OCT) is a low-loss, visible, non-invasive imaging technique that uses low-coherence light to obtain cross-sectional images of samples by measuring the interference signals reflected from different depths. While OCT has been widely used in biomedical fields, such as ophthalmology, dermatology and cardiology, its application to solid-state battery research is relatively new. As shown in Figure 1b,c, OCT can provide valuable information about the morphology and growth of lithium dendrites at different operating conditions, such as current density, temperature, and cycle number. Additionally, OCT can also reveal the effects of various design parameters, such as electrolyte composition and anode thickness, on the dendrite behavior.

In this paper, we present a comprehensive study of lithium dendrites at the interface of solid-state batteries based on OCT. We perform OCT imaging of the battery interface during cycling under different conditions and analyze the OCT images to extract quantitative information about dendrite morphology and growth. Furthermore, we compare the OCT results with SEM results to verify the accuracy and effectiveness of the OCT method. We believe that the OCT method provides essential experimental and theoretical support for studying the electrochemical reaction mechanism, performance degradation, and failure of SSBs.

## 2. Experiments

### 2.1. Principles of OCT Characterization of Solid-State Batteries

The OCT technology was initially applied to ophthalmology to recognize eye diseases in their early stages. In this study, we first introduced OCT to the research of in situ characterization of solid-state batteries. The OCT optical imaging system, which is based on optical coherence tomography, can provide both two-dimensional and three-dimensional images of solid-state batteries in real time. This capability contributes to a deeper understanding of the mechanism in the working process of batteries. The OCT system used in this study was based on spectral-domain OCT (SD-OCT), which consists of a broadband light source, a fiber-optic Michelson interferometer, a spectrometer, and a two-dimensional scanning device.

In this study, SD-OCT is used for real-time in situ monitoring of solid-state batteries. Solid-state batteries use common polymers as solid-state electrolytes, lithium sheets for positive and negative electrodes, and transparent organic glass and silicone grease for packaging materials to ensure that the OCT scanning light enters the interior of the solid-state battery. Before monitoring, it is necessary to use OCT scanning to obtain the initial state of the solid-state battery. When the solid-state battery experiences a cycle alarm, scanning detection is performed to obtain dendrite morphology, and continuous scanning is performed to obtain dendrite growth during the cycle.

SD-OCT is composed of a low temporal coherence light source, a Michelson interferometer, a spectrometer, a reference mirror, and a transverse scanning device. The core components of SD-OCT are the Michelson interferometer and the spectrometer. The spectrometer consists of a collimating lens, a diffraction grating, an imaging lens, and a CCD line scan camera. The light from the light source is coupled into a 2 × 2 fiber-optic coupler, which enters the reference arm with mirrors and the sample arm with the solid-state battery under test, respectively. The reference light reflected by the mirror and the backscattered signal light of the solid-state battery is recombined by the fiber coupler to generate an interference signal. The interference signal is output from the other end of the fiber coupler, received by the spectrometer, and expanded by the diffraction grating in the spectrometer. The signal is then collected by the CCD, converted into an electrical signal, digitized, and input to the computer. The obtained data is subjected to inverse Fourier transform to obtain the depth information of the solid-state battery. Finally, through transverse scanning, the two-dimensional tomographic image of the solid-state battery can be reconstructed, thereby obtaining the structure of the solid-state battery under test.

### 2.2. Preparation of Solid Electrolyte

**Figure 2:**
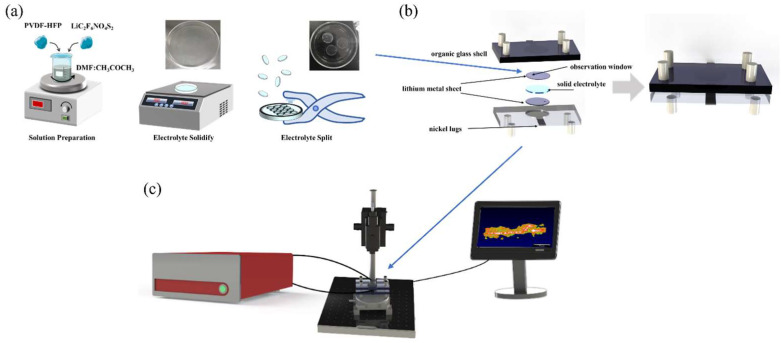
(**a**) Preparation of solid electrolyte. (**b**) Assembly of solid-state batteries. (**c**) Schematic diagram of in situ characterization of solid-state batteries by OCT.

The preparation of solid electrolyte can be divided into four steps, as shown in Figure 2a. Firstly, the solid electrolyte used in the solid-state battery was a polymer electrolyte. The main materials used were polyvinylidene fluoride-hexafluoropropylene copolymer (PVDF-HFP) and lithium salt, lithium bis (trifluoromethane sulfonyl) imide (LiC_2_F_6_NO_4_S_2_) (mass ratio: PVDF-HFP:LiC_2_F_6_NO_4_S_2_ = 1:1.5). Secondly, the electrolyte material was dissolved in a mixed solution of 1:3 volume ratio of N, N-dimethylformamide (DMF) and acetone (CH_3_COCH_3_) and stirred with a magnetic stirrer until the electrolyte was completely dissolved into the mixed solution. Thirdly, the solution of the electrolyte material was evenly poured into a petri dish, and then placed on a heating plate, heated to 70 °C for 2 h to solidify the electrolyte. Finally, the solidified electrolyte was cut with a puncher to match the shape of the electrode (area = 1.9 cm^2^). The thickness of the lithium metal electrode is 5 μm (China Energy Lithium Company, Tianjin, China). The thickness of the solid electrolyte is 40 μm.

### 2.3. Assembly of Solid-State Batteries

As shown in Figure 2b, the solid-state battery mainly consists of a non-conductive organic glass shell, lithium metal sheet positive and negative electrodes, solid electrolyte, and nickel lugs. The transparent organic glass shell ensures that the light from OCT can penetrate the shell, enabling real-time observation of the battery’s internal working state. To prevent oxidation of the lithium electrode, the assembly of solid-state batteries must be carried out in a glove box filled with argon. First, the positive electrode, a lithium metal sheet, is placed in a custom-made organic glass shell. These shells feature a circular groove with a depth of 600 μm, matching the diameter of the lithium metal sheet. Secondly, the solid electrolyte cut to the same diameter was placed on top of the lithium metal sheet. Thirdly, another lithium metal sheet was placed on top of the solid electrolyte to serve as a negative electrode. Finally, the positive and negative electrodes were connected with nickel lugs and encapsulated with silicone grease to prevent the oxidation of metallic lithium.

### 2.4. In Situ Characterization of Solid-State Batteries by OCT

The packaged solid-state battery was positioned in the optical coherence tomography (OCT) imaging system. The battery was charged and discharged via an electrochemical workstation to monitor its operational status, as depicted in Figure 2c. The OCT optical imaging system is employed for continuous scanning of the solid-state battery to realize real-time in situ monitoring of the state of the battery. Firstly, the height of the sample arm of the OCT was adjusted to ensure that the solid–solid interface of the solid-state battery could be clearly observed. Secondly, the battery was charged and discharged with a constant current of 1.9 mA (the current density is 1 mA/cm^2^), the deposition time of the charge and discharge was 1 h, and the interval was 5~15 min. Thirdly, when the battery I–V curve fluctuated greatly or was unstable, the OCT was used to scan the battery to observe changes in the internal state of the battery in real time, and record the morphology of lithium dendrites inside the battery, which could contribute to a better understanding of the internal situation of the battery. Finally, the current was reduced to 0.4 mA, and OCT was used for continuous scanning to achieve continuous characterization of the lithium dendrite growth at the solid–solid interface inside the solid-state battery, as show in the videos in the Supplementary Materials.

## 3. Results and Discussions

### 3.1. Dendrite Growth Image Based on OCT Imaging System

**Figure 3:**
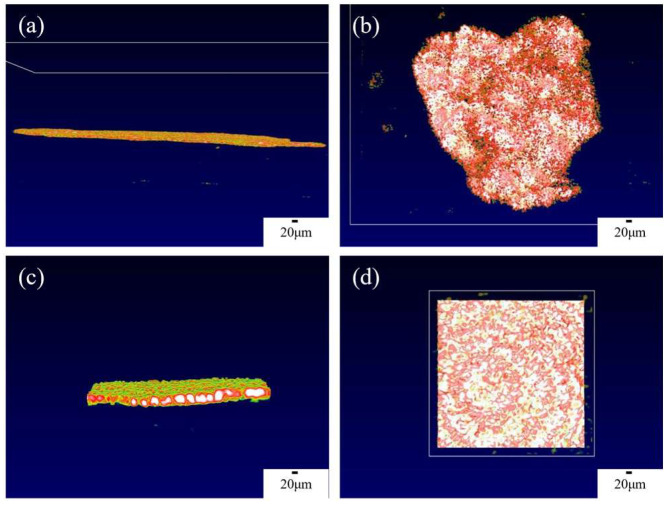
OCT images of the internal interface of solid-state batteries from different angles. (**a**) Overall side view of the interface, (**b**) overall top view of the interface, (**c**) 200 × 200 μm side view, (**d**) 200 × 200 μm top view.

The OCT imaging system enables the characterization of the solid-interface shape of solid-state batteries and the appearance of lithium dendrite growth, thereby providing a clearer and more comprehensive analysis of lithium dendrite growth. Figure 3a,b present the overall side view and top view of the interface window, respectively. In order to obtain higher quality results, a smaller field of view can be used to scan the solid-state battery, which will be more helpful in analyzing the growth of lithium dendrites, as shown in Figure 3c,d, which depict a 200 × 200 μm random area of the overall side view and top view, respectively. From Figure 3, the interface is flat in the initial state at each angle, so the growth of the lithium dendrites can be determined by analyzing changes in the interface. The color of OCT in this article is mainly related to temperature. Due to the ion transport at the interface between the positive and negative electrodes of the battery and the electrolyte during the charge and discharge process, the interface temperature is high. Therefore, we can find that the solid electrolyte at higher temperatures appears white and red, while the electrode appears green.

**Figure 4:**
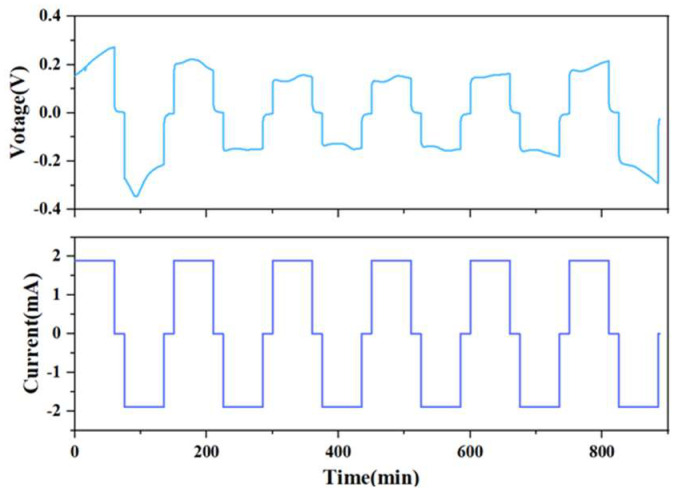
Solid-state battery charge and discharge test I-V curve.

In order to observe the change of the internal solid interface during the charging and discharging process of the battery, the current is set at 1.9 mA, the deposition time is 1 h, the interval is 15 min, and the alarm voltage is at ±3 V. From Figure 4, it can be seen that in the first five cycles, although the I-V curve of the solid-state battery fluctuates slightly, the overall shape is relatively uniform and has a battlement pattern, indicating that the charge–discharge cycle process of the battery is relatively stable.

**Figure 5:**
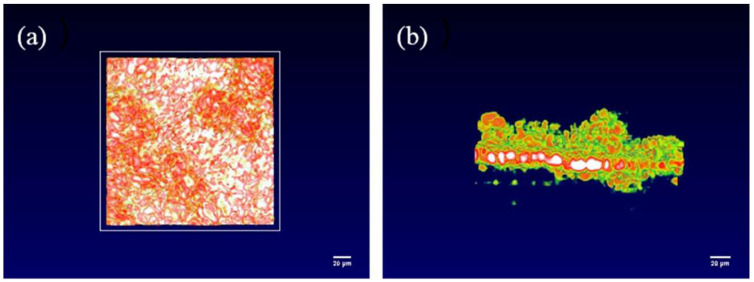
OCT image of the dendrite structure of the solid-state battery. (**a**) Top view of the interface, (**b**) side view of the interface.

From Figure 5, it could be seen that the OCT image of the battery solid interface was obtained by the OCT. Compared with the original state of the solid battery interface, it can be clearly observed that there are protrusions of varying heights on the solid interface. These represent lithium dendrites of different heights and shapes that appeared during the deposition. In addition, the uneven distribution of color depth in the top view indicates that the surface of the solid interface has become uneven due to the emergence of lithium dendrites. This fully demonstrates that the OCT optical imaging system can be used as a new method for the in situ characterization of solid-state batteries. OCT can not only clearly and comprehensively characterize the changes of lithium dendrites during the entire operation of solid-state batteries, but also obtain the final morphology inside the battery.

### 3.2. Dendrite Growth Analysis

**Figure 6:**
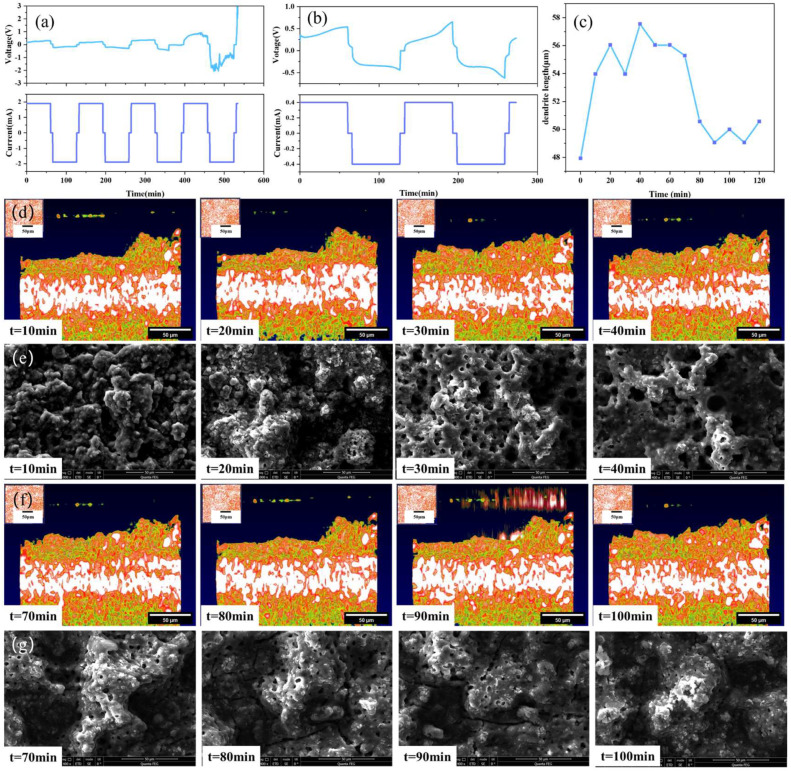
Solid-state battery test results: (**a**) 1.9 mA constant current test, (**b**) 0.4 mA constant current test, (**c**) dendrite growth state diagram, (**d**) OCT images of variation trend of dendrites in the stage of battery charging, (**e**) SEM images of variation trend of dendrites in the stage of battery charging, (**f**) OCT images of variation trend of dendrites in the stage of battery discharging, (**g**) SEM images of variation trend of dendrites in the stage of battery discharging.

Compared with traditional characterization methods, the advantage of the OCT optical imaging system is that it could not only observe the initial and final states of solid-state batteries, but also observe and analyze the dynamic evolution process of the dendrite growth of the electrolyte during the charging and discharging cycles. Figure 6 shows the test results of the solid-state battery sample. A constant current of 1.9 mA is used to test the charge and discharge cycle of the battery, as shown in Figure 6a. The results show that the voltage profiles of the batteries exhibit a battlement shape, which is typical for solid-state batteries with lithium metal electrodes. The battlement shape is caused by the polarization and relaxation processes of the electrodes and the electrolyte during charging and discharging. The polarization process is related to the charge transfer resistance and the diffusion resistance of lithium ions in the electrodes and the electrolyte, while the relaxation process is related to the redistribution and recombination of lithium ions in the electrodes and the electrolyte. The polarization and relaxation processes are affected by the current density, cycle number, temperature, and other factors.

After the voltage surge triggers an alarm, the morphology of the lithium dendrites is observed by OCT. The current is then reduced to 0.4 mA to continue the charge and discharge cycles, and OCT is used to continuously scan and obtain a set of lithium dendrite growth state evolution diagrams, as shown in Figure 6b. The results show that the voltage profiles of the batteries become more irregular and unstable with increasing current density and cycle number, which is mainly due to the formation and growth of lithium dendrites at the electrode/electrolyte interface.

As shown in Figure 6c, in order to further understand the growth process of lithium dendrites, the change in the highest point of lithium dendrites at the peak of the subsequent charge and discharge cycles was measured using ImageJ, with the peak value of the protrusion after the fifth charge and discharge cycle as a reference point. It can be seen from Figure 6c that the height of the bulge in the charging stage is generally greater than that in the discharge stage. This is because the working principle of the solid-state battery involves the repeated movement of lithium ions between the positive and negative electrodes. When the battery is being charged, lithium ions are deintercalated from the positive electrode and embedded into the negative electrode through the solid-state electrolyte. The negative electrode is in a lithium-rich state, causing the lithium dendrites at the interface between the negative electrode and the solid-state electrolyte to be more prominent and protruding. Conversely, during the discharge process, lithium ions are released from the negative electrode and move back to the positive electrode. Due to the deintercalation movement of lithium ions, the lithium dendrites at the interface of the negative electrode contract and become less prominent. At the same time, since the battery itself is solid, the interface between the electrolyte and the electrode is a solid–solid interface, which is different from the spinel-like lithium dendrites of liquid lithium-ion batteries. There is mechanical rigidity and high pressure between the solid–solid interface, and the lithium dendrites tend to be smooth and flat, which also verifies the accuracy of lithium dendrite growth. Figure 6d,e show the OCT images and SEM images depicting the variation trend of dendrites during the battery charging stage. Figure 6e,f show the OCT images and SEM images depicting the variation trend of dendrites during the battery discharging stage. The changes in lithium dendrites can be clearly seen from the SEM images during the charge and discharge process. The experimental measurement results of OCT in Figure 6 are consistent with the theoretical analysis results, thus proving the good potential of OCT for in situ characterization of the solid-state battery operating process.

**Figure 7:**
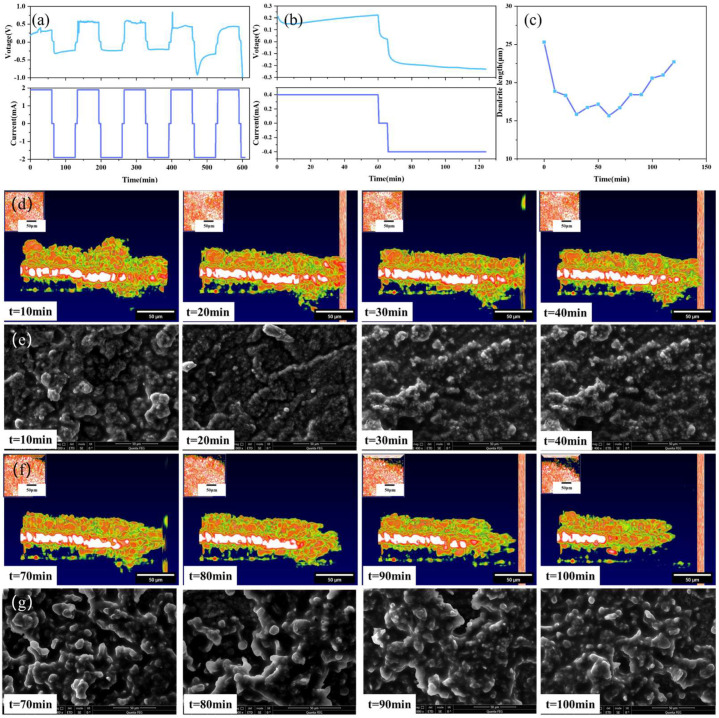
Solid-state battery test results: (**a**) 1.9 mA constant current test, (**b**) 0.4 mA constant current test, (**c**) dendrite growth state diagram, (**d**) OCT images of variation trend of dendrite in the stage of battery charging, (**e**) SEM images of variation trend of dendrite in the stage of battery charging, (**f**) OCT images of variation trend of dendrite in the stage of battery discharging, (**g**) SEM images of variation trend of dendrite in the stage of battery discharging.

In order to more comprehensively and clearly understand the dendrite growth process of solid-state batteries, multiple batteries were assembled for experimental testing in this research. During charging, lithium ions move to the interface of the perforated lithium sheet, and during discharging, the opposite occurs. The experimental results are shown in Figure 7.

Figure 7a,b depict the I–V curves of the solid-state battery at different currents. After the fourth charge-discharge cycle, an obvious convex structure at the interface can be clearly observed by the OCT imaging system. Figure 7c shows the growth process of the interface dendrites for one cycle after the current is adjusted to 0.4 mA. As shown in Figure 7d,e, the height of the dendrite has a process of decreasing and increasing, which is because in this experiment, the positive and negative electrodes are reversed. During charging, the negative electrode loses lithium ions, resulting in a decrease in the height of the dendrites formed by the deposition of lithium ions. On the contrary, during discharge, the negative electrode is rich in lithium, and the height of lithium dendrites has a process of increasing.

### 3.3. Verification and Comparison of SEM Images

**Figure 8:**
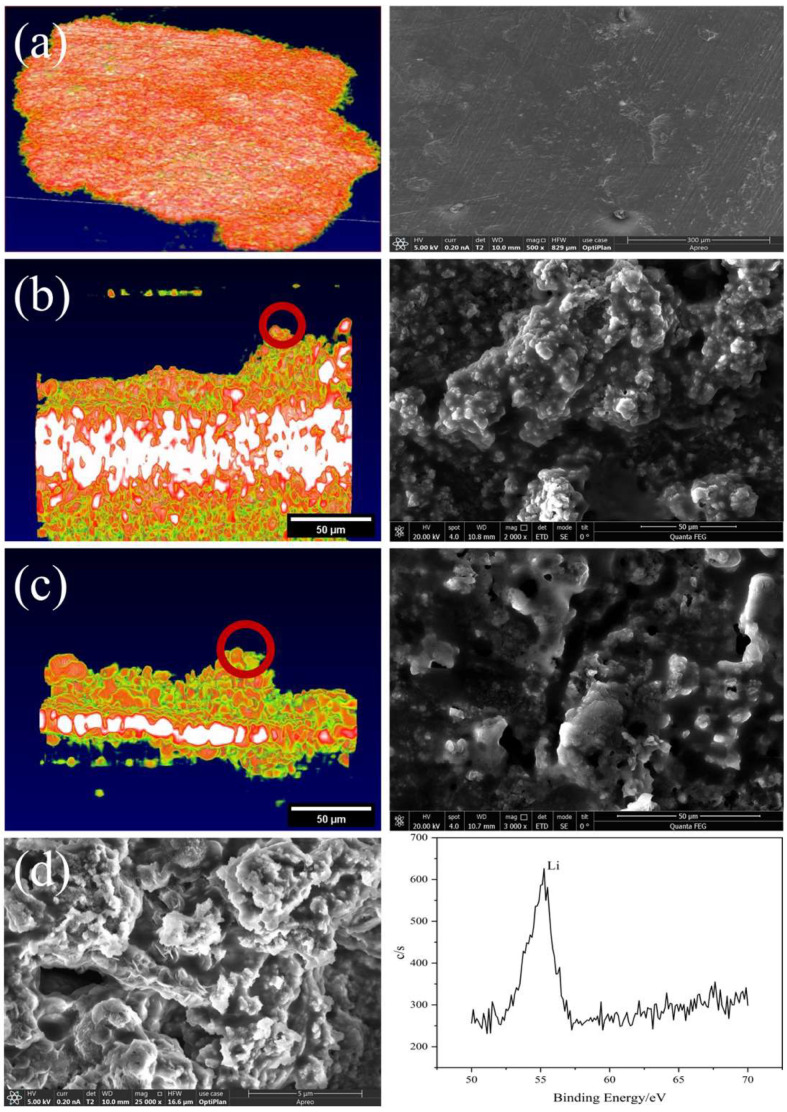
Characterization of solid-state batteries: (**a**) the OCT and SEM of the lithium metal electrode in its initial state, (**b**) OCT and SEM images showing dendrite formation after charging Battery 1, (**c**) OCT and SEM images showing dendrite formation after charging Battery 2, (**d**) SEM and XPS characterization of lithium dendrites.

In order to further verify that the bulge on the interface between the battery electrode and the solid electrolyte is the lithium dendrite formed by the irregular deposition of lithium, as shown in Figure 8, SEM was used to characterize the battery. The comparison between the electrochemical performance and OCT/SEM images of the batteries demonstrates that the OCT method can provide valuable information for understanding the electrochemical reaction mechanism, performance degradation, and failure in solid-state batteries. In addition, to characterize the protrusions as lithium dendrites, we further performed X-ray Photoelectron Spectroscopy (XPS). The XPS results showed the existence of lithium dendrites and verified the accuracy of the experimental record.

The results show that the voltage profiles of the batteries become more irregular and unstable with increasing current density and cycle number, which is mainly due to the formation and growth of lithium dendrites at the electrode/electrolyte interface, as observed by OCT and SEM. From both OCT and SEM, it can be clearly seen that the interface has obvious fluctuations, indicating that deposition forms lithium dendrites. In addition, the lithium dendrites in the OCT images are smooth, which is also consistent with the actual SEM images. Through the characterization of SEM and XPS, it can be further confirmed that the appearance and growth state of lithium dendrites on the solid–solid interface of solid-state batteries are highly consistent with the OCT characterization results. The comparison between the electrochemical performance and OCT/SEM images of the batteries demonstrates that the OCT method can provide valuable information for understanding the electrochemical reaction mechanism, performance degradation, and failure in solid-state batteries, which is a prospective new method that can be used for in situ characterization of solid-state batteries.

## 4. Conclusions

In this paper, we propose a new method based on OCT for the in situ characterization of the internal state and lithium dendrite growth of solid-state batteries. The prepared solid-state batteries were assembled using a polymer as the solid electrolyte and lithium metal as positive and negative electrodes. The OCT system characterized the batteries before and after undergoing various cycles at different current densities. We validated the accuracy and effectiveness of the OCT method by comparing the OCT images with SEM and XPS. The OCT method can image the internal structure and lithium dendrite growth of solid-state batteries in situ clearly and effectively, without causing any damage or interference. It provides valuable information for understanding the electrochemical reaction mechanism, performance degradation, and failure in solid-state batteries, such as the formation and evolution of lithium dendrites, voids, and cracks at the electrode–electrolyte interface. Additionally, the OCT method can aid in optimizing the design and fabrication of solid-state batteries by offering feedback on the impact of various parameters, such as current density, cycle number, temperature, electrode material, and electrolyte material, on the internal state and lithium dendrite growth. The OCT method is a powerful tool for characterizing solid-state batteries and other energy materials and devices. The OCT method has several advantages over conventional methods, such as being non-invasive, non-destructive, real-time, and visible. The OCT method can also be combined with other techniques, such as electrochemical impedance spectroscopy, Raman spectroscopy, etc., to obtain more comprehensive and complementary information. The OCT method has great potential for future research and development of solid-state batteries and other energy materials and devices.

## Figures and Tables

**Table 1:** Comparison of different characterization techniques.

Methods	Features
Optical microscopy	Optical microscopy is a non-contact and non-vacuum analysis tool that places fewer restrictions on in situ cell design. However, it has certain limitations for interface analysis.
SEM	SEM can provide better spatial resolution than optical microscopy but not as good as TEM. However, SEM needs to be carried out under vacuum conditions.
Neutron depth profiling/ Neutron tomography	Since the scattering cross-section of neutrons is complementary to that of X-rays and electrons, neutron-based characterization techniques offer unique advantages. Neutrons are more sensitive to light atoms (such as H, Li, O) compared to X-rays/electrons and have the ability to distinguish between neighboring elements. Neutrons have strong penetrating properties, which is beneficial to non-destructive in situ/operational measurements of batteries. However, NPD can accurately provide 3D atomic-level structural information, but only for battery materials with good crystallinity and long-range structural order.
NMR/MRI	Metal casings etc. are usually present in batteries, but batteries used for in situ measurements must have as few metal parts as possible. This will have limitations on battery manufacturing.
Atomic force/ Scanning probe microscopy	Scanning probe microscopy can form high spatial resolution surface images by scanning a physical probe on a sample, and it can operate in a variety of environments, such as liquids or electrochemical environments. However, it is a surface-sensitive technology, and its application is limited to the characterization of battery electrodes or electrolyte surface properties.
TEM	TEM can pass an electron beam through a thin sample to form images with ultra-high spatial resolution. However, to maximize resolution, TEM operations require a high vacuum environment.
Synchrotron tomography	Synchrotron-based X-ray imaging technology is a powerful tool for battery research and can probe a variety of length scales, with varying depth sensitivities and spatial/temporal resolutions. Operational experiments enable the characterization of battery charging and discharging processes.
XRD	XRD can obtain information about the chemical and structural properties of electrode materials and the electrochemical mechanism. However, obtaining information on amorphous and nanomaterials is challenging.
Neutron diffraction	Neutron diffraction generally has an advantage over its X-ray counterpart because of its greater sensitivity to the location of moving species (light ions such as Li+ and H+) inside the battery. Compared with commonly used ex situ experiments, in situ characterization through neutron diffraction can provide a more comprehensive understanding of the structural processes of electrodes. In addition, X-ray tomography can also be combined to achieve three-dimensional imaging of the inside of the battery, which will help to gain a deeper understanding of the internal working mechanism of the battery.
OCT	OCT technology is a new method that can be used for solid-state batteries, which can achieve the same results as traditional characterization methods. With the continuous deepening of research on the application of OCT technology in the battery field, OCT technology is expected to become a new method that can be used for battery monitoring.

## Data Availability

Data are contained within the article.

## Supplementary Materials

The following supporting information can be downloaded at: https://www.mdpi.com/article/10.3390/s24082392/s1, Video S1: OCT solid electrolyte interface characterization 1. Video S2: OCT solid electrolyte interface characterization 2.
